# Deep learning-based cervical cancer T-staging using MRI: multi-structure segmentation and classification

**DOI:** 10.1186/s12880-026-02449-2

**Published:** 2026-05-30

**Authors:** Shanshan Xu, Yuxin Zou, Zhe Wu, Zhou Xu, Ruiwei Wang, Wenjing Hou, Yanzhou Wang, Yi Wu

**Affiliations:** 1 Department of Digital Medicine, College of Biomedical Engineering and Medical Imaging, Army Medical University, (Third Military Medical University), Chongqing, 400038 China; 2 Department of Medical Engineering of Jiangbei Campus, The First Affiliated Hospital of Army Medical University (The 958th hospital of Chinese PLA), Chongqing, 400020 China; 3 Yu-Yue Pathology Research Center, Jinfeng Laboratory, Chongqing, 401329 China; 4 Department of Radiology, The First People’s Hospital of Neijiang, Neijiang, Sichuan Province 641000 China; 5 Department of Oncology, Zigong First People’s Hospital, Zigong, Sichuan Province 643000 China; 6 Department of Radiology, First Affiliated Hospital of Army Medical University, Third Military Medical University), Chongqing, 400038 China; 7 Department of Obstetrics and Gynecology, Chongqing, 400038 China

**Keywords:** Cervical cancer, MRI, Deep learning, Segmentation, T-staging diagnosis

## Abstract

**Aims:**

Cervical cancer has high incidence and mortality, seriously threatening women’s survival and quality of life. Radiologists currently rely mainly on subjective clinical experience for cervical cancer T-staging, which easily leads to misdiagnosis.

**Objectives:**

To develop a deep learning-based technique for automatic segmentation and T-staging of cervical cancer to improve clinical diagnostic accuracy and efficiency.

**Materials and methods:**

A dataset of 17,479 fT1WI MRI scans from 144 patients (T1–T4 stages) was constructed; tumors and adjacent structures were manually outlined with Amira 2019 to generate ground truth (GT). A novel segmentation network (CPANet) was designed by integrating global pyramid guidance (GPG) and atrous spatial pyramid pooling (ASPP) modules into CNN. CPANet’s segmentation performance was validated against GT and compared with UNet, UNet++, DeepLabv3+, and UperNet-Swin. Based on CPANet-extracted ROIs, T-staging models were built using ResNet50, DenseNet121, and Swin Transformer (pathological T-staging as GT), and the optimal model was selected.

**Results:**

CPANet outperformed other networks, with Dice similarity coefficients (DSCs) of 0.783 (tumor), 0.901 (uterus), 0.909 (bladder), and 0.892 (rectum), and average per-case processing time of 1.60 s. Swin Transformer achieved the best T-staging performance: AUCs of 0.713 (T1), 0.799 (T2), 0.845 (T3–T4) for main stages, and 0.623 (T1b), 0.673 (T2a), 0.897 (T2b) for sub-stages from MRI images. This not only improves diagnostic accuracy and efficiency but also conserves medical resources, thereby facilitating the establishment of intelligent healthcare systems in medically underserved areas.

**Conclusions:**

CPANet and Swin Transformer enable accurate automatic segmentation and T-staging of cervical cancer from MRI images, improving diagnostic accuracy and efficiency, saving medical resources, and facilitating intelligent healthcare in underserved areas.

## Introduction

Cervical cancer ranks as the fourth most commonly diagnosed cancer among women in the United States, with approximately 13,820 new cases and 4,280 deaths projected for 2026 [1, 2]. Given its profound impact on women’s health and quality of life, timely detection, early diagnosis, and prompt treatment constitute critical determinants of improved prognosis [3, 4] and prolonged survival [5]. Accurate T-staging is critical for treatment planning, as early-stage patients typically undergo surgical intervention while advanced-stage cases require chemoradiotherapy [6]. Pelvic MRI serves as the gold standard for local tumor staging [7], and deep learning-based image analysis has emerged as a promising approach for automated segmentation and classification [8]. Despite advances in deep learning for medical imaging, systematic investigations integrating multi-structure segmentation and T-staging classification for cervical cancer using MRI remain limited [9]. This study proposes a deep learning-based framework combining CPANet for multi-structure segmentation and Swin Transformer for T-staging classification using MRI.

The rapid advancement of deep learning has enabled extensive applications in cervical cancer diagnostics. Various algorithms, including YOLO [10], ResNet [11, 12], and VGG [13–15], have demonstrated high diagnostic performance (accuracy exceeding 97%) [16]. For instance, Yuan et al. developed a ResNet-based model have shown AUC = 0.93 for colposcopic diagnosis, and Lin YC et al. [17] reported that U-Net has achieved Dice = 0.82 for cervical tumor segmentation in MRI. However, existing studies predominantly focus on cytopathological images and binary classification tasks [18]. Systematic investigations integrating multi-structure segmentation and T-staging classification for cervical cancer using MRI remain limited [19].

Consequently, this study established a segmentation dataset based on fat-suppressed T1-weighted imaging (fT1WI) images, encompassing cervical tumors and adjacent structures. We designed a segmentation network (CPANet) incorporating global pyramid guidance (GPG) and atrous spatial pyramid pooling (ASPP) modules. Leveraging regions of interest (ROIs) extracted from CPANet’s segmentation outputs, we developed and compared T-staging diagnostic models using three representative deep learning architectures—ResNet50, DenseNet121, and Swin Transformer—to identify the optimal predictor for cervical cancer T-stages.

## Materials and methods

This retrospective study was compliant with the Standards for Reporting of Diagnostic Accuracy guidelines and was approved by the institutional review board of Army Medical University (B-KY2024067) and Zigong First People’s Hospital (2023-Research No. 111). Given that clinical data were acquired during routine clinical care, the requirement for informed consent was waived.

### Study population

**Fig. 1:**
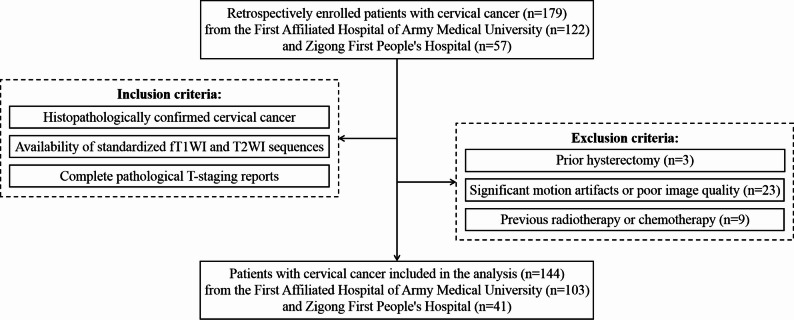
Patient inclusion and exclusion flowchart

A total of 179 cervical cancer patients were retrospectively enrolled from two participating centers: the First Affiliated Hospital of Army Medical University (Center 1, November 2020–March 2022) and Zigong First People’s Hospital (Center 2, September 2020–February 2023). Inclusion criteria included: (1) histopathologically confirmed cervical cancer; (2) availability of standardized fT1WI and T2WI sequences; and (3) complete pathological T-staging reports. Exclusion criteria comprised: (1) prior hysterectomy (*n* = 3); (2) significant motion artifacts or poor image quality (*n* = 23); and (3) previous radiotherapy or chemotherapy (*n* = 9). Finally, 144 patients included in the analysis, distributed across T1-T4 stages (T1: 55, T2: 56, T3: 29, T4: 4). Figure 1 illustrates the patient selection workflow, and Tables 1 and 2 provides patient clinical information data and grouping of this study.

**Table 1:** Patient clinical information

Hospital	T1	T2	T3	T4	Training set	Validation set	Test set	Total
XN Hospital	40	41	19	3	72 (8682)	10 (1215)	21 (2605)	103 (12502)
ZG Hospital	15	15	10	1	28 (3456)	4 (484)	9 (1037)	41 (4977)
Total	55	56	29	4	100 (12138)	14 (1699)	30 (3642)	144 (17479)

The data format is the number of patients (images)

**Table 2:** Data grouping of T-staging diagnosis of cervical cancer (cases)

Class	Training set	Validation set	Test set	Total
Main stage grouping				
T1	39	5	11	55
T2	38	6	12	56
T3-T4	23	3	7	33
Total	100	14	30	144
Sub stage grouping				
T1b	37	5	10	52
T2a	25	4	7	36
T2b	14	2	4	20
Total	76	11	21	108

The data format is the number of patients

### Image acquisition and MRI protocol

**Fig. 2:**
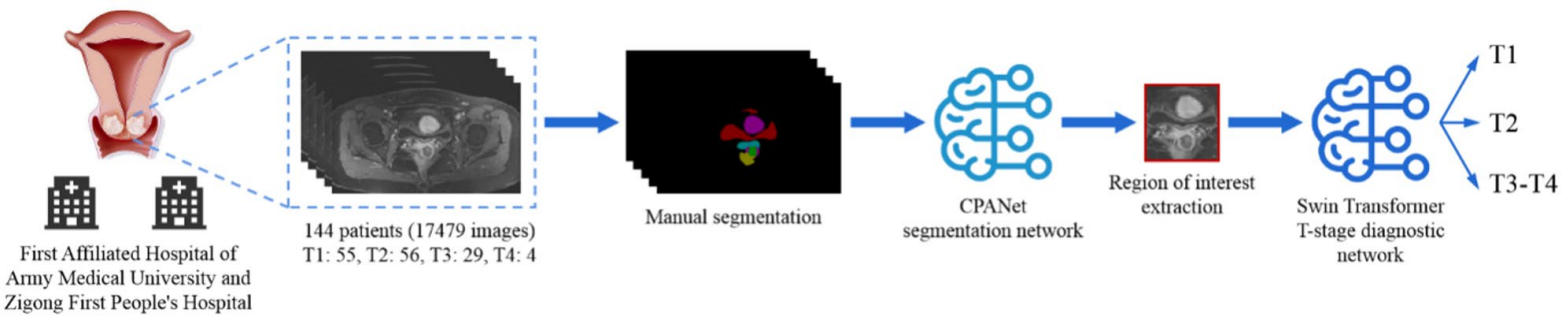
The overall workflow of this study

**Fig. 3:**
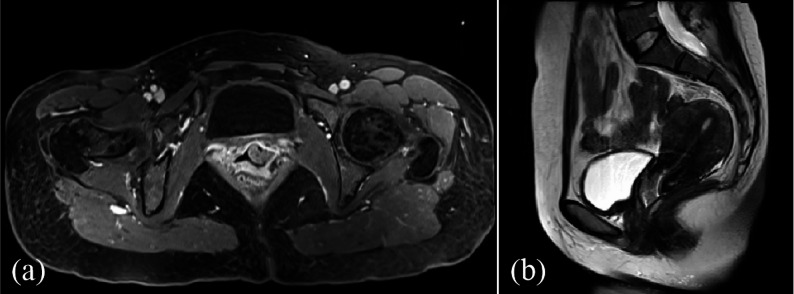
Representative MRI images: (**a**) axial fat-suppressed T1-weighted imaging and (**b**) sagittal T2-weighted imaging

The overall workflow of this study is illustrated in Fig. 2. MRI examinations were performed at two institutions using 3.0T Siemens MR Scanners (Siemens Healthineers, Erlangen, Germany) with turbo spin-echo (TSE) sequences. Axial fT1WI served as the primary sequence for tumor segmentation, while sagittal T2-weighted imaging (T2WI) was used for anatomical reference (Fig. 3).

All imaging data were retrieved from institutional PACS systems in DICOM format. fT1WI parameters: Center 1 (TR 4.86 ms, TE 1.81 ms, flip angle 10°, matrix 768 × 456, slice thickness 1 mm) and Center 2 (TR 2.88 ms, TE 1.13 ms, flip angle 9°, matrix 320 × 260, slice thickness 1.2 mm). T2WI parameters: Center 1 (TR 4000 ms, TE 101 ms, matrix 320 × 320, slice thickness 5 mm) and Center 2 (TR 4060 ms, TE 89 ms, matrix 432 × 432, slice thickness 4 mm). All imaging data were retrieved in DICOM format from institutional PACS platforms.

### Manual segmentation and 3D reconstruction

**Fig. 4:**
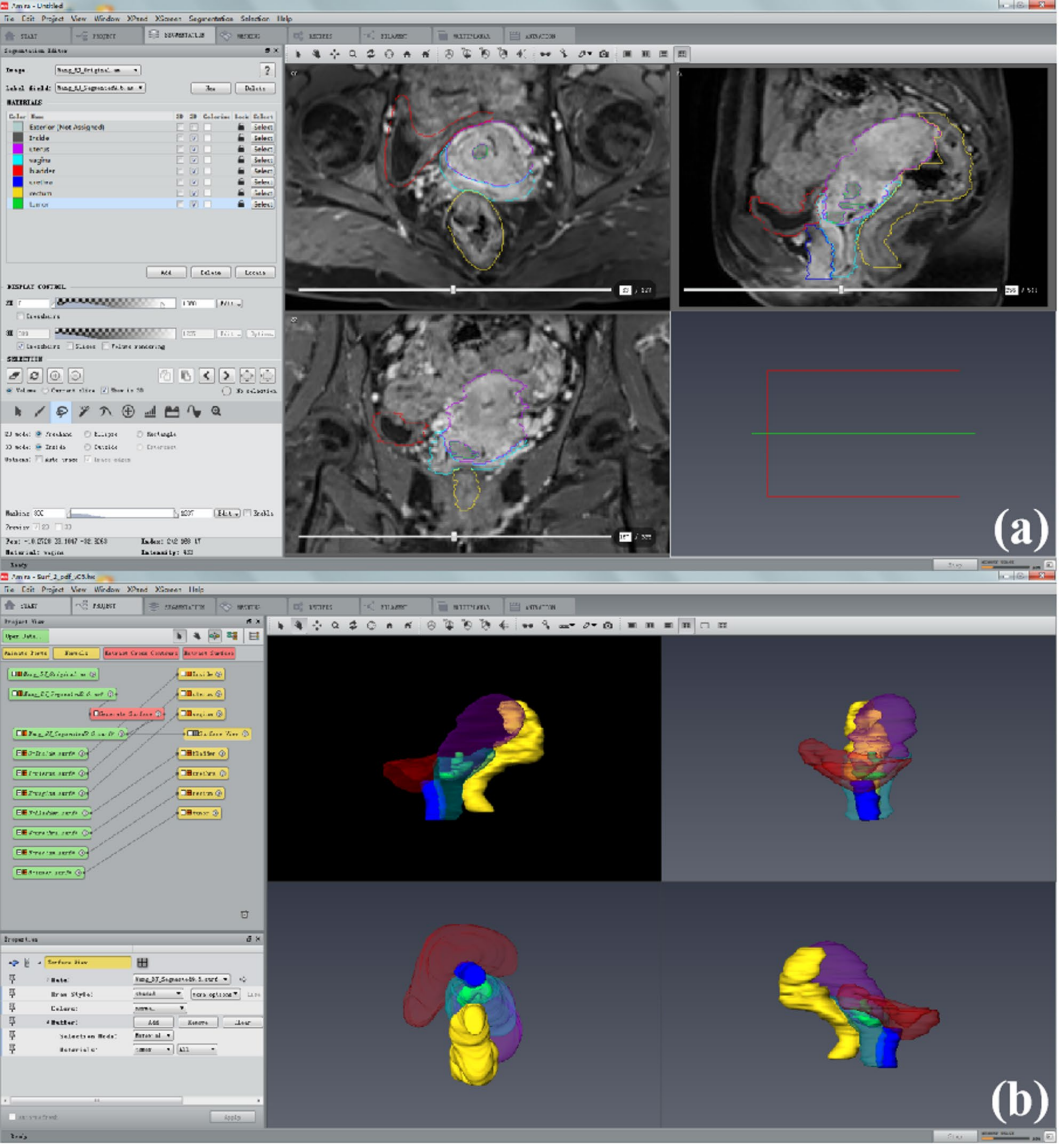
Manual segmentation (**a**) and 3D reconstruction interface (**b**) of Amira software

Manual segmentation was performed using Amira software (Version 2019, Thermo Fisher Scientific Inc., and Waltham, MA, USA) with optimized window width/level settings to delineate six anatomical structures: cervical tumor, uterus, vagina, bladder, urethra, and rectum. Segmentation criteria were based on signal intensity differences and anatomical boundaries observed in MRI sequences. Cervical carcinoma typically appeared hypointense on fT1WI and hyperintense on T2WI sequences. All segmentation was independently performed by a junior radiologist and reviewed by a senior radiologist. Discrepancies were resolved by a third senior radiologist (Fig. 4a).

For 3D reconstruction, non-constrained smoothing algorithm was applied using Amira’s Generate Surface module to perform surface rendering from axial segmentation masks. Post-reconstruction processing included mesh smoothing and simplification to optimize visualization while preserving anatomical fidelity. The resulting 3D models enabled multiplanar manipulation (translation, scaling, and rotation) for comprehensive spatial analysis (Fig. 4b).

### Construction and evaluation of segmentation models

#### Model architecture and training

**Fig. 5:**
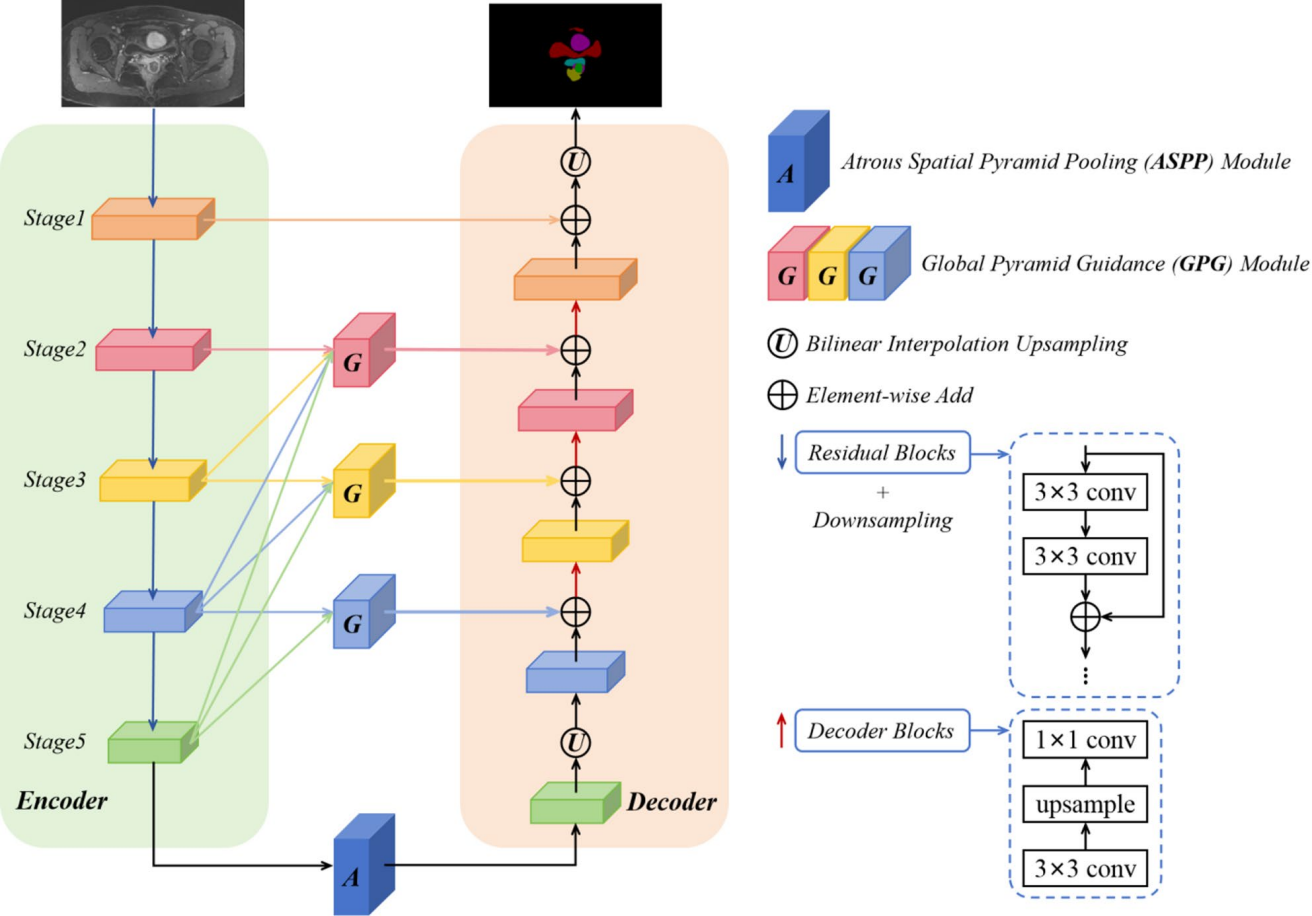
Structure diagram of the designed CPANet

We designed CPANet, a novel U-shaped encoder-decoder network for medical image segmentation, incorporating two key modules: the global pyramid guidance (GPG) module [19] and the atrous spatial pyramid pooling (ASPP) module [20] (Fig. 5). The GPG modules are placed between encoder and decoder to fuse global contextual information, while the ASPP module at the encoder top captures multi-scale features.

Preprocessing included: DICOM-to-CT conversion, windowing (width = 1000, level = 450), normalization, and resampling to 320 × 320 pixels. Data augmentation (random erasing, rotation, flipping, brightness/contrast adjustment) was applied to address limited training data. Five deep learning networks—UNet [21], UNet++ [22], DeepLabv3+ [23], UperNet-Swin [24], and CPANet—were trained and compared using our custom segmentation dataset as ground truth.

#### Experimental setup

Training was performed using PyTorch 1.10.1 on Python 3.7.1, with an NVIDIA Quadro RTX A4000 GPU (16 GB VRAM). Models were optimized with Adam optimizer, cross-entropy loss, initial learning rate of 0.001, 100 epochs, and batch size of 16. Optimal weights were selected based on minimum validation loss, with final evaluation on the test set.

#### Evaluation metrics

This study employed the dice similarity coefficient (DSC) as the primary metric for evaluating segmentation model performance, supplemented by additional indicators including intersection over union (IoU), Precision, Recall, F_1_-score, and 95% Hausdorff distance (95%HD). Additionally, segmentation times for the five models and manual segmentation were recorded on the test set to facilitate comparative analysis of computational efficiency. The mathematical formulations for these metrics are defined as follows:

1 $$\:DSC=\frac{2|X\cap\:Y|}{\left|X\right|+\left|Y\right|}$$

2 *\:IoU=TP/(TP+FP+FN)*

3 *\:Precision=TP/(TP+FP)*

4 *\:Recall=TP/(TP+FN)*

5 $$\eqalign{ {F_1} - score = & {2 \over {{1 \over {Precision}} + {1 \over {Recall}}}} \cr& = {{2TP} \over {FP + 2TP + FN}} \cr}$$

6 $$\eqalign{ HD & = {d_H}\left( {X,Y} \right) = max\left\{ {{d_{XY}},{d_{YX}}} \right\} \cr& = max\left\{ {\mathop {\max }\limits_{x \in X} \mathop {\min }\limits_{y \in Y} d\left( {x,y} \right),\mathop {\max }\limits_{y \in Y} \mathop {\min }\limits_{x \in X} d\left( {x,y} \right)} \right\} \cr}$$

#### Ablation study

To validate the individual contributions of each module in CPANet, we conducted ablation experiments by progressively removing key modules for comparative analysis. Specifically, we designed three experimental configurations: (1) Baseline, the standard UNet without ASPP and GPG modules; (2) UNet + ASPP, which adds the atrous spatial pyramid pooling module on the basis of Baseline; and (3) UNet + ASPP + GPG, the complete CPANet architecture with both modules integrated. Through this progressive module addition approach, we can clearly observe the performance improvements contributed by each module.

### Construction and evaluation of T-staging diagnostic model

#### Swin transformer architecture and model training

**Fig. 6:**
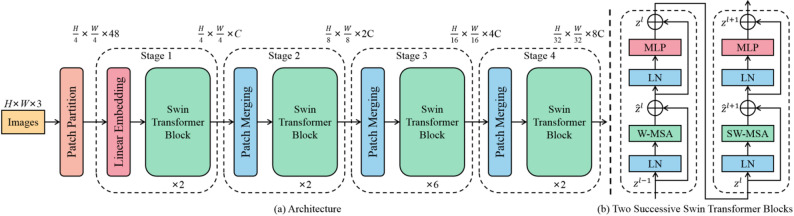
Swin Transformer architecture: (**a**) overall structure; (**b**) successive Swin Transformer Blocks. W-MSA: window-based multi-head self-attention; SW-MSA: shifted-window multi-head self-attention

We constructed a cervical cancer T-staging diagnostic model based on the Swin Transformer architecture (Fig. 6) [25]. The model processes input images through patch partitioning, Linear Embedding, and hierarchical Patch Merging. Each stage consists of stacked Swin Transformer Blocks with window-based MSA (W-MSA) for efficiency and shifted-window MSA (SW-MSA) for cross-window exchange. Three representative networks—ResNet50 [26], DenseNet121 [27], and Swin Transformer—were trained using pathological T-stages as ground truth. ROIs extracted from CPANet segmentation were preprocessed and input into all networks for T-staging prediction.

The experimental environment and hyperparameters were consistent with those described in Sect.  2.4.1, except for using stochastic gradient descent (SGD) as the network optimizer. SGD computes gradients and updates model parameters using either single samples or mini-batches per iteration, offering advantages of computational simplicity and real-time updates. Training parameters included an initial learning rate of 0.0001, 100 epochs, and a batch size of 8.

#### T-staging diagnostic performance evaluation metrics

This study employed confusion matrices and receiver operating characteristic (ROC) curves as primary metrics for evaluating T-staging diagnostic model performance, supplemented by additional indicators including Precision, Recall, Specificity, F_1_-score, Accuracy, and area under curve (AUC). The formulations for metrics are provided below:

7 $$\:\mathrm{S}\mathrm{p}\mathrm{e}\mathrm{c}\mathrm{i}\mathrm{f}\mathrm{i}\mathrm{c}\mathrm{i}\mathrm{t}\mathrm{y}=\mathrm{T}\mathrm{N}/(\mathrm{T}\mathrm{N}+\mathrm{F}\mathrm{P})$$

8 $$\:\mathrm{A}\mathrm{c}\mathrm{c}\mathrm{u}\mathrm{r}\mathrm{a}\mathrm{c}\mathrm{y}=(\mathrm{T}\mathrm{P}+\mathrm{T}\mathrm{N})/(\mathrm{T}\mathrm{P}+\mathrm{T}\mathrm{N}+\mathrm{F}\mathrm{P}+\mathrm{F}\mathrm{N})$$

The confusion matrix is structured with True labels as the x-axis and Predicted labels as the y-axis. Each row represents all samples predicted for a specific class, with diagonal values indicating correctly classified instances. Greater concentration of values along the diagonal signifies superior model classification performance.

The ROC curve plots 1-Specificity (x-axis) against Sensitivity (y-axis), where closer proximity to the top-left corner indicates better model performance. The AUC value, representing the area under the ROC curve, ranges from 0 to 1, with higher values indicating superior classification capability.

### statistical analysis

Statistical significance of differences in segmentation performance (DSC, IoU, Precision, Recall, F1-score, 95%HD) among the five models was evaluated using the Friedman test, followed by post-hoc Wilcoxon signed-rank test with Bonferroni correction for pairwise comparisons. Similarly, the Friedman test was applied to compare classification performance metrics (Precision, Recall, Specificity, F1-score, Accuracy, AUC) among the three T-staging models, with post-hoc pairwise comparisons. The statistical significance level was set at *p* < 0.05. All statistical analyses were performed using Python 3.7.1 with SciPy 1.7.0.

## Results

### Performance of CPANet-based segmentation model

Quantitative segmentation results for CPANet and comparative models on the test set are presented in Table 3. Except for the vaginal structure where UNet + + achieved the highest DSC, the proposed CPANet model demonstrated the highest DSC values across the remaining five structures (tumor: 0.783, uterus: 0.901, bladder: 0.909, urethra: 0.885, rectum: 0.892). Furthermore, CPANet exhibited superior overall performance across all evaluation metrics including IoU, Precision, Recall, F1-score, and 95%HD.

**Table 3:** Quantitative segmentation results for CPANet and comparative models on the test set

Metrics	Models	Segmentation structures					
		uterus	vagina	bladder	urethra	rectum	tumor
Dice similarity coefficient	UNet++	0.856	**0.792**	0.903	0.871	0.878	0.782
	DeepLabv3+	0.732	0.508	0.622	0.652	0.701	0.675
	UperNet-Swin	0.838	0.769	0.897	0.855	0.865	0.760
	CPANet	**0.901**	0.790	**0.909**	**0.885**	**0.892**	**0.783**
Precision	UNet++	0.886	0.758	0.879	0.867	0.897	0.735
	DeepLabv3+	0.775	0.560	0.621	0.679	0.660	**0.779**
	UperNet-Swin	0.872	0.734	0.872	0.852	0.886	0.710
	CPANet	**0.923**	**0.758**	**0.887**	**0.882**	**0.909**	0.736
Recall	UNet++	0.827	**0.828**	0.927	0.874	0.860	0.834
	DeepLabv3+	0.694	0.465	0.622	0.627	0.747	0.596
	UperNet-Swin	0.807	0.807	0.922	0.858	0.846	0.818
	CPANet	**0.879**	0.825	**0.931**	**0.887**	**0.876**	**0.837**
F_1_-score	UNet++	0.856	**0.792**	0.903	0.871	0.878	0.782
	DeepLabv3+	0.732	0.508	0.621	0.652	0.701	0.675
	UperNet-Swin	0.838	0.769	0.897	0.855	0.865	0.760
	CPANet	**0.901**	0.790	**0.909**	**0.885**	**0.892**	**0.783**
95% hausdorff distance (mm)	UNet++	18.358	13.429	22.558	4.198	11.724	15.413
	DeepLabv3+	32.504	25.230	37.334	11.919	22.488	27.476
	UperNet-Swin	19.978	13.942	23.126	4.307	12.377	16.167
	CPANet	**16.786**	**12.344**	**20.099**	**3.684**	**10.575**	**13.137**

**Fig. 7:**
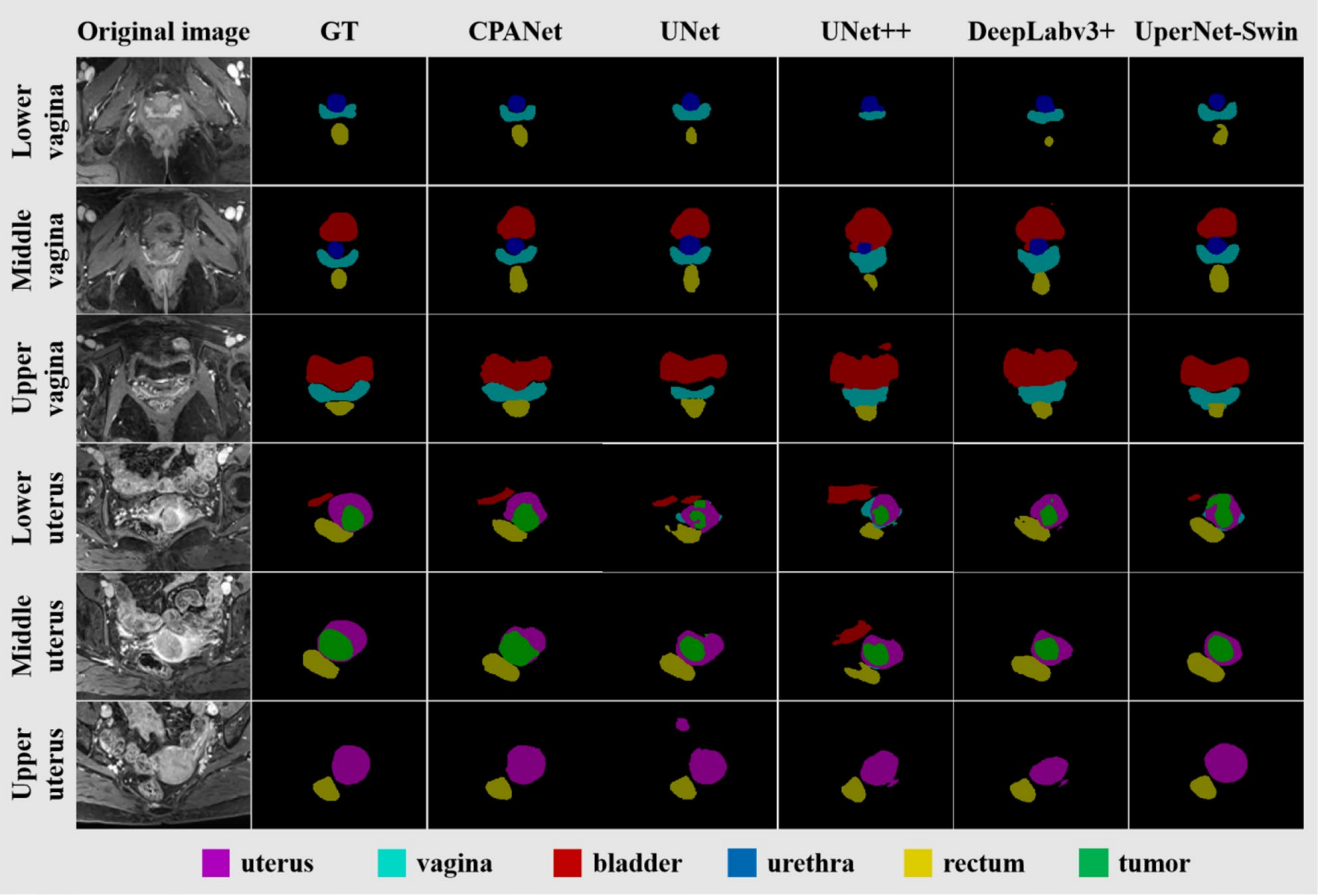
Visual comparison of segmentation results of CPANet and other models with GT

The segmentation results of the proposed CPANet model demonstrated the highest similarity to GT compared to other networks. For cervical tumors and adjacent structures of varying sizes and shapes, the CPANet model demonstrated excellent segmentation performance in delineating their contour boundaries (Fig. 7).

The ablation experimental results demonstrate that with the progressive integration of the ASPP module and GPG module, CPANet exhibits consistent improvements in Dice coefficient, Precision, Recall, and F1-score across all target tissues, while the 95% HD progressively decreases, indicating that both modules effectively enhance the segmentation performance of the model. Notably, the complete CPANet achieves optimal results in the segmentation of bladder (0.909) and rectum (0.892), among other tissues (Table 4).

**Table 4:** Ablation experiment

Metrics	Models	Regions of interest					
		uterus	vagina	bladder	urethra	rectum	tumor
DSC	Unet	0.842	0.769	0.897	0.855	0.867	0.764
	Unet + ASPP	0.867	0.780	0.903	0.870	0.875	0.770
	UNet + ASPP + GPG (CPFNet)	**0.901**	**0.790**	**0.909**	**0.885**	**0.892**	**0.783**
Precision	Unet	0.875	0.733	0.872	0.851	0.888	0.715
	Unet + ASPP	0.880	0.749	0.879	0.866	0.895	0.725
	UNet + ASPP + GPG (CPFNet)	**0.923**	**0.758**	**0.887**	**0.882**	**0.909**	**0.736**
Recall	Unet	0.812	0.809	0.923	0.858	0.848	0.820
	Unet + ASPP	0.834	**0.831**	0.917	0.860	0.864	**0.842**
	UNet + ASPP + GPG (CPFNet)	**0.879**	0.825	**0.931**	**0.887**	**0.876**	0.837
F1-score	Unet	0.842	0.769	0.897	0.855	0.867	0.764
	Unet + ASPP	0.844	**0.801**	0.903	0.867	0.881	**0.785**
	UNet + ASPP + GPG (CPFNet)	**0.901**	0.790	**0.909**	**0.885**	**0.892**	0.783
95% HD (mm)	Unet	19.781	14.575	24.034	4.505	12.751	16.506
	Unet + ASPP	19.015	13.119	23.438	4.607	11.564	16.660
	UNet + ASPP + GPG (CPFNet)	**16.786**	**12.344**	**20.099**	**3.684**	**10.575**	**13.137**

For each patient, the average time of manual segmentation was 5424.73s, the UNet + + took the shortest time of 1.19s in all models, the UperNet-Swin took the longest time of 1.81s, the CPANet took the middle time of 1.60s (Table 5).

**Table 5:** Average segmentation time between models and manual methods on the test set

	Manual segmentation	CPANet	UNet	UNet++	DeepLabv3+	UperNet-Swin
Time (s)	5424.73$\:\:\pm\:$52.68	1.60$\:\:\pm\:\:$0.14	1.50$\:\pm\:\:$0.14	1.19$\:\pm\:\:$0.09	1.40$\:\pm\:\:$0.13	1.81$\:\pm\:\:$0.16

### Performance of swin transformer-based cervical cancer T-staging diagnostic model

Quantitative results for T-staging prediction by the Swin Transformer and comparative models on the test set are presented in Table 6. The Swin Transformer model achieved an accuracy of 0.7 in predicting cervical cancer T1, T2, and T3-T4 stages, with AUC values of 0.713, 0.799, and 0.845 for T1, T2, and T3-T4 staging, respectively. These results demonstrate superior performance compared to ResNet50 and DenseNet121. Additionally, the Swin Transformer model exhibited the highest overall performance across all evaluation metrics including Precision, Recall, Specificity, and F_1_-score. Similarly, for the T1b, T2a, and T2b sub-staging classification scheme, the Swin Transformer model also achieved the best overall performance across all evaluation metrics.

**Table 6:** Quantitative results of deep learning models in predicting Tumor staging

Models	Precision	Recall	Specificity	F_1_-score	AUC	Accuracy
Main stage grouping						
ResNet50						0.600
T1	0.571	0.364	0.842	0.444	0.579	
T2	0.550	0.917	0.500	0.688	0.748	
T3-T4	1	0.429	1	0.600	0.773	
DenseNet121						0.600
T1	0.600	0.545	0.789	0.571	0.667	
T2	0.600	0.750	0.667	0.667	0.778	
T3-T4	0.600	0.429	0.913	0.500	0.767	
Swin Transformer						0.700
T1	0.700	0.636	0.842	0.667	0.713	
T2	0.643	0.750	0.722	0.692	0.799	
T3-T4	0.833	0.714	0.957	0.769	0.845	
Sub stage grouping						
ResNet50						0.619
T1b	0.714	0.500	0.818	0.588	0.591	
T2a	0.545	0.857	0.643	0.667	0.719	
T2b	0.667	0.500	0.941	0.571	0.801	
DenseNet121						0.476
T1b	0.571	0.400	0.727	0.471	0.586	
T2a	0.444	0.571	0.643	0.500	0.653	
T2b	0.400	0.500	0.824	0.444	0.787	
Swin Transformer						0.571
T1b	0.714	0.500	0.818	0.588	0.623	
T2a	0.444	0.571	0.643	0.500	0.673	
T2b	0.600	0.750	0.882	0.667	0.897	

**Fig. 8:**
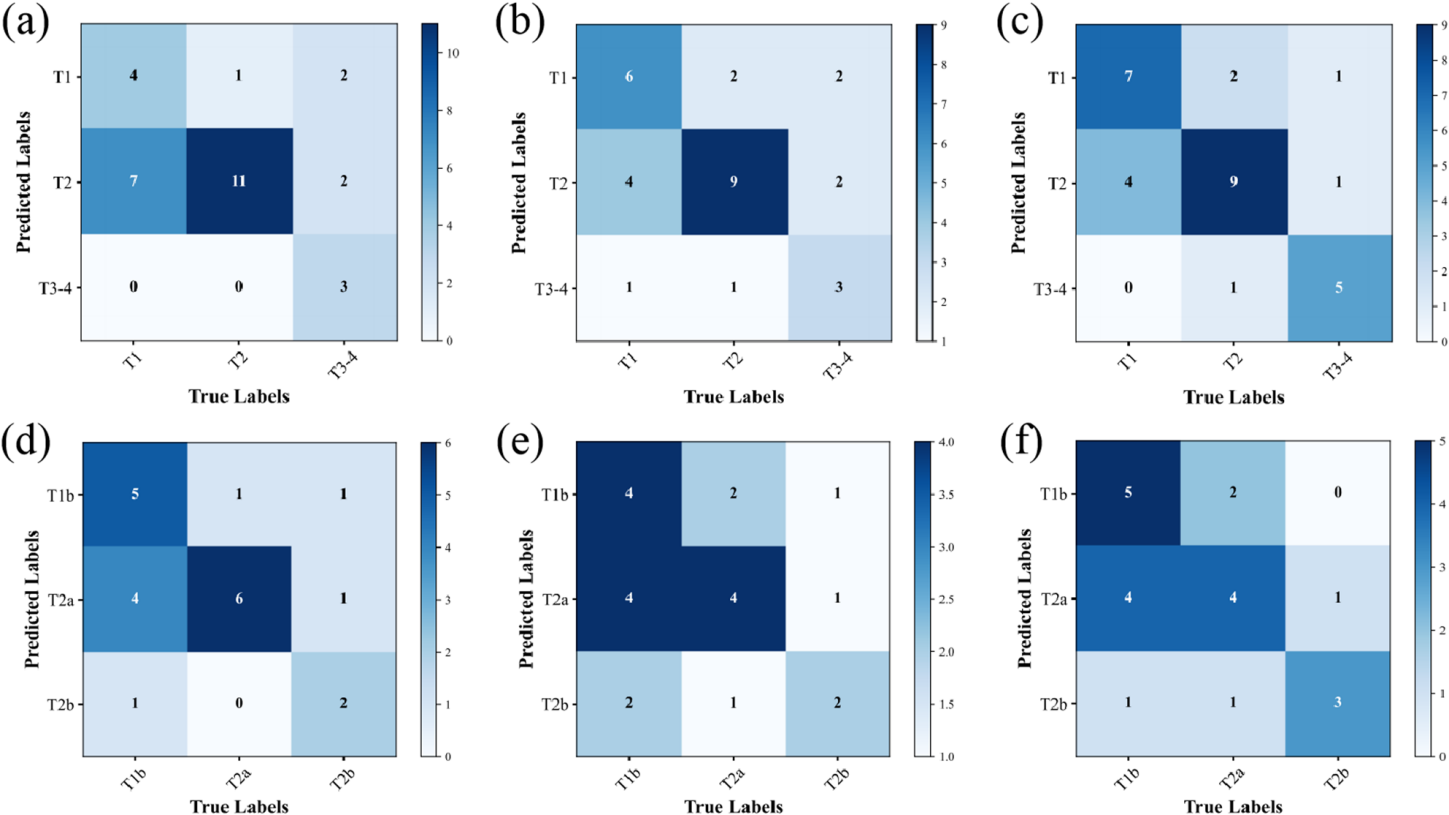
Confusion matrix for predicting T-staging diagnosis of cervical cancer using three models. (**a**-**c**) confusion matrix for T1, T2, T3-T4. (**d**-**f**) confusion matrix for T1b, T2a, T2b. (**a** and **d**) ResNet50. (**b** and **e**) DenseNet121. (**c** and **f**) Swin Transformer

**Fig. 9:**
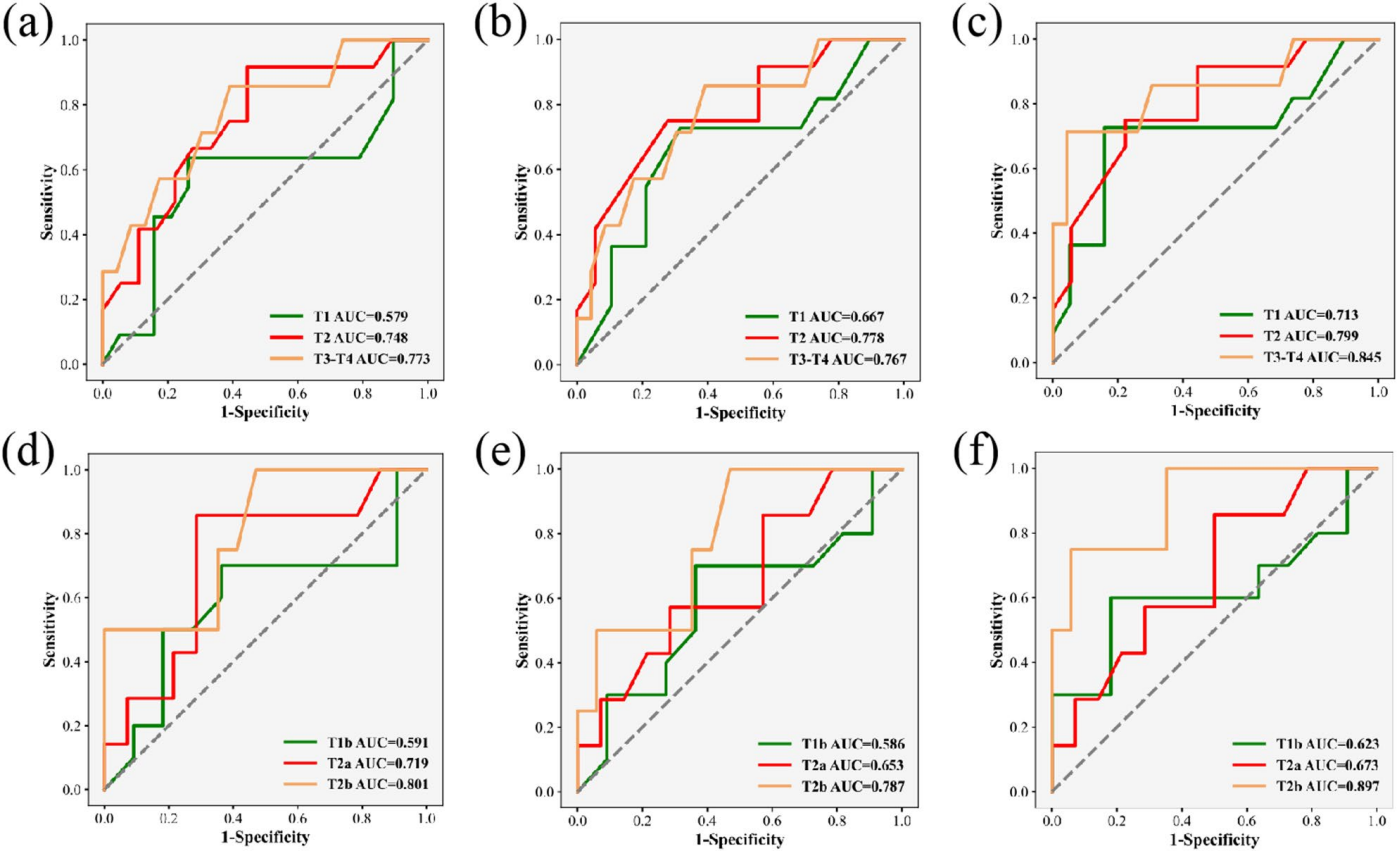
ROC curves for predicting T-staging diagnosis of cervical cancer using three models. (**a**-**c**) ROC curve for T1, T2, T3-T4. (**d**-**f**) ROC curve for T1b, T2a, T2b. (**a** and **d**) ResNet50. (**b** and **e**) DenseNet121. (**c** and **f**) Swin transformer

The confusion matrix and ROC curves for T-staging prediction by the three models are illustrated in Figs. 8 and 9, respectively. For the three-class classification task (T1, T2, T3-T4), the Swin Transformer model demonstrated the highest total number of correctly classified cases along the confusion matrix diagonal, although ResNet50 achieved slightly better performance specifically for T2 classification. In the three-class sub-staging task (T1b, T2a, T2b), ResNet50 showed the highest total number of correct diagonal classifications, while the Swin Transformer model outperformed others in T2b classification accuracy.

**Fig. 10:**
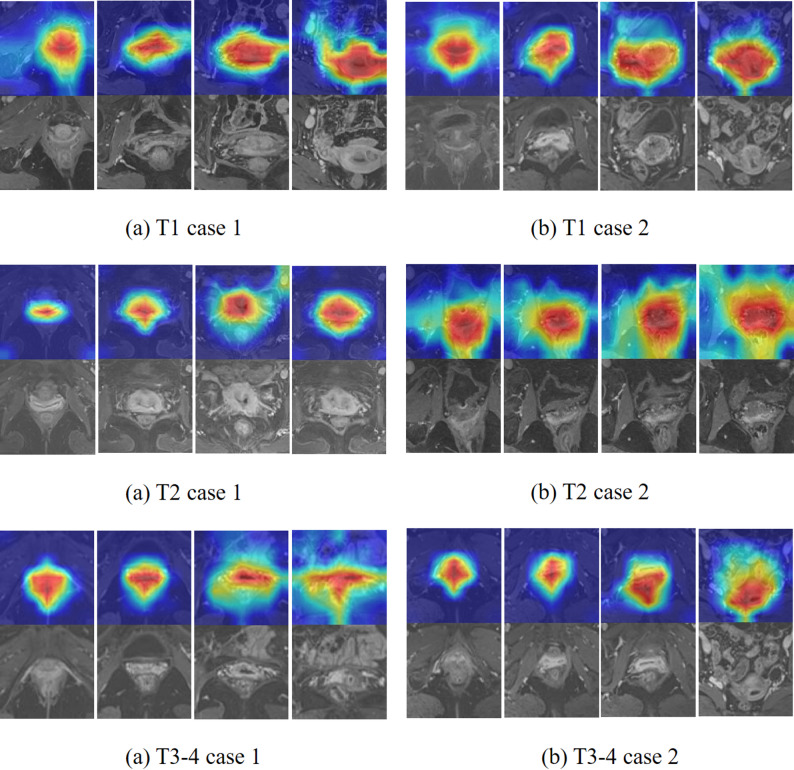
Class activation mapping of Swin Transformer model on test set

We selected six patients from the test set, including two cases each for T1, T2, and T3-T4 stages, and generated class activation mapping (CAM) visualizations for the Swin Transformer model using the widely adopted Grad-CAM [28] algorithm (Fig. 10). As demonstrated, the model’s attention areas on the test set primarily concentrated around tumor regions and adjacent structures. This observation confirms that the Swin Transformer model effectively focuses on clinically relevant regions critical for T-staging diagnosis, aligning with the diagnostic attention patterns of radiologists. This spatial correlation explains the superior T-staging diagnostic performance achieved by the Swin Transformer model.

## Discussion

Cervical cancer remains one of the most prevalent malignant tumors among women globally, with early detection and precise diagnosis critical for improving survival and quality of life. Medical imaging plays a pivotal role in diagnosis, staging, and treatment planning. However, raw imaging data alone often fails to comprehensively evaluate tumor extent. Image segmentation enhances interpretability by delineating tumours and adjacent structures, supporting clinical staging decisions.

Notably, segmentation performance varied across anatomical levels even within the same model architecture. At the levels of lower, middle, and upper vagina, as well as the upper uterus, the model achieved segmentation results closely approximating GT for the urethra, bladder, vagina, upper uterus, and rectum, with only minor protrusions or indentations presenting challenges for precise segmentation. However, at the levels of lower and middle uterus, the model exhibited lower segmentation accuracy for tumor, the uterus-vagina junction, and the middle uterus, resulting in ambiguous boundaries between structures. These findings align with the observed difficulties in manual segmentation of these specific anatomical regions.

In recent years, deep learning has been increasingly applied in cervical cancer analysis. For segmentation tasks, Wubineh et al. [29] developed a multi-network framework for cytoplasm and nuclei segmentation in cervical cytology images, achieving 99.02% precision using EfficientNetB2. Zhu et al. [30] proposed CoTr, a hybrid CNN-Transformer for cervical tumor MRI segmentation, achieving DSC = 0.827. Ma et al. [31] introduced VB-Net for clinical target volume delineation in cervical cancer radiotherapy. While existing studies have achieved notable progress, most focus on single-structure or cellular segmentation, primarily involving OARs in CT images for brachytherapy planning [29, 31]. To our knowledge, no studies have explored simultaneous segmentation of tumors and six adjacent structures in MRI. Building upon CNN architectures, we developed CPANet by integrating GPG and ASPP modules.

To address these limitations, we developed CPANet, a novel U-shaped network integrating GPG and ASPP modules for simultaneous segmentation of cervical tumors and six adjacent structures. Based on CPANet segmentation outputs, we constructed T-staging diagnostic models using three representative architectures (ResNet50, DenseNet121, Swin Transformer), establishing an integrated pipeline for comprehensive cervical cancer analysis.

Quantitative results demonstrated that CPANet achieved the highest DSC values for 5 of 6 anatomical structures (tumor: 0.783, uterus: 0.901, bladder: 0.909, urethra: 0.885, rectum: 0.892), with processing time of only 1.60 s (3,390× faster than manual segmentation). The Swin Transformer model achieved the best T-staging performance (accuracy: 0.70; AUC: 0.713/0.773/0.797 for T1/T2/T3-T4). Class activation mapping confirmed that Swin Transformer focused predominantly on tumor regions and adjacent structures, aligning with radiologists’ diagnostic patterns.

The superior performance of CPANet is attributed to: (1) the GPG module facilitating global context fusion with decoder features; and (2) the ASPP module capturing multi-scale contextual information for structures of varying sizes. The Swin Transformer’s success stems from its hierarchical window-based self-attention architecture, efficiently capturing both local and global imaging features critical for T-staging.

To our knowledge, this is the first study to simultaneously segment cervical tumors and six adjacent structures in MRI using a unified deep learning framework. Compared to existing studies limited to single-structure segmentation [18, 30, 31] or cytopathological binary classification [17, 19, 32–34], our integrated pipeline provides comprehensive multi-structure analysis and T-staging classification, demonstrating clear superiority in both scope and clinical applicability.

### Limitations

This study has several limitations. First, the dataset size was relatively small with uneven distribution across T-stages. Second, CPANet’s vaginal segmentation performance lagged behind UNet++. Third, data were from only two institutions. Future work will focus on dataset expansion and external validation.

## Conclusions

The CPANet and Swin Transformer models developed in this study can achieve rapid and accurate auto-segmentation of cervical tumors and adjacent structures in MRI, as well as precise T-staging diagnosis. These models have the potential to improve clinical diagnostic accuracy and efficiency, reduce workflows, and facilitate intelligent healthcare development.

## Data Availability

The datasets used and analyzed during the current study are available from the corresponding author on reasonable request.

## Abbreviations

ASPP: Atrous spatial pyramid pooling
AUC: Area under receiver operating characteristic curve
CTVs: Clinical target volumes
DSCs: Dice similarity coefficients
DWI: Diffusion weighted image
GPG: Global pyramid guidance
GT: Ground truth
IoU: Intersection over union
MLP: Multilayer perceptrons
OARs: Organs at risk
ROC: Receiver operating characteristic
SGD: Stochastic gradient descent
SVM: Support vector machines
TNM: Tumor-node-metastasis
95%HD: 95% Hausdorff distance
